# Copper nanowire embedded hypromellose polymer: An antibacterial nanocomposite film

**DOI:** 10.1016/j.jcis.2021.09.130

**Published:** 2021-09-25

**Authors:** Biswajoy Bagchi, Carmen Salvadores Fernandez, Manni Bhatti, Lena Ciric, Laurence Lovat, Manish K. Tiwari

**Affiliations:** 1 Wellcome/EPSRC Centre for Interventional and Surgical Sciences (WEISS), University College London, London, W1W 7TS, UK; 2 Nanoengineered Systems Laboratory, UCL Mechanical Engineering, University College London, London, WC1E 7JE, UK; 3 UCL Department of Civil, Environmental and Geomatic Engineering, London, WC1E 6BT, UK

**Keywords:** Copper nanowire, Antimicrobial, Hypromellose film, Biocompatibility

## Abstract

The present work reports a novel antibacterial nanocomposite film comprising of copper nanowire impregnated biocompatible hypromellose using polyethylene glycol as a plasticiser. Detailed physico-chemical characterization using X-ray diffraction, Fourier transform infrared spectroscopy, UV-Visible spectroscopy and electron microscopy shows uniform dispersion of copper nanowire in the polymer matrix without any apparent oxidation. The film is flexible and shows excellent antibacterial activity against both Gram positive and negative bacteria at 4.8 wt% nanowire loading with MIC values of 400 μg/mL and 500 μg/mL for *E. coli* and *S. aureus* respectively. Investigation into the antibacterial mechanism of the composite indicates multiple pathways including cellular membrane damage caused by released copper ions and reactive oxygen species generation in the microbial cell. Interestingly, the film showed good biocompatibility towards normal human dermal fibroblast at minimum bactericidal concentration (MBC). Compared to copper nanoparticles as reported earlier *in vitro* studies, this low cytotoxicity of copper nanowires is due to the slow dissolution rate of the film and production of lower amount of ROS producing Cu^2+^ ions. Thus, the study indicates a strong potential for copper nanowire-based composites films in broader biomedical and clinical applications.

## Introduction

Bacterial adhesion and proliferation in trauma and fracture related wounds pose a serious healthcare concern with regards to developing strategies for combating nosocomial infections. Extensive use of antibiotics has led to emergence of pathogenic drug-resistant strains for both Gram negative and positive bacteria which are difficult to control and eradicate [1, 2]. This has prompted researchers worldwide to find alternatives in the form of novel antibacterial agents and composites [3]. To this end, metal and metal oxide-based nanostructures have been shown to have potent antibacterial activity due to their unique physico-chemical attributes such as size, surface charge and solubility. These nanomaterials can penetrate the bacterial membrane causing physical and biochemical damage through multiple pathways without any risk of inducing antimicrobial resistance [3–4]. For example, gold, silver and copper nanoparticles exhibit strong microbicidal activities against a wide variety of pathogenic bacterial and fungal strains [5–10]. Among these, copper-based antimicrobials have gained widespread popularity as a cost-effective alternative with high biological activity and comparatively lower ecological concern [10]. Furthermore, humans have been shown to tolerate copper to a reasonable extent, thereby encouraging potential application as bactericidal bandages and coatings on medical devices [11–16]. The only disadvantage is, in contrast to noble metals, copper nanoparticles tend to oxidise very quickly and hence long-term efficacy is affected [10]. Thus, inclusion of copper nanoparticles in a suitable biocompatible matrix is emerging as a common strategy to form stable antibacterial nanocomposites. For instance, in one of the early studies, Cioffi *et al*. [11], incorporated copper nanoparticles in polyvinylmethylketone, poly-(vinyl chloride) and polyvinylidene fluoride and studied their bactericidal and antifungal properties. Later, copper nanoparticles were introduced into different matrices such as polyvinyl alcohol, chitosan, cellulose, polyester, alginate, clays, hydrogels and polyimide RO membrane, which showed strong long-term bactericidal and antifungal activity [17–26]. In all these works, the polymer matrix played a vital role in both protecting the nanoparticles from oxidation as well as enabling sustained release.

Although copper is an important trace mineral in the human body, there is still concern regarding the cytotoxicity of copper nanoparticles on human cells [11–14]. The cytotoxicity mainly arises from the small size and spherical shape of nanoparticles employed which facilitates easy penetration and consequently leads to direct cell membrane damage, protein and DNA agglomeration and oxidative stress inside the cell by ROS generation [11–16]. As an alternative, we propose to exploit copper nanowires (CuNWs). It is interesting to evaluate the bioactivity of CuNWs because of their diverse ligand dependent surface chemistry [27] and markedly greater effective size than spherical particles which should influence their cell penetrability [28]. This will open a way to mitigate the cytotoxicity associated with spherical copper nanoparticles while keeping the antimicrobial properties intact. Copper nanowires have a widespread application in the electronic industry due to their high electrical conductivity which enables their exploitation in the manufacturing of conducting sheets, sensors and flexible electrodes [29]. Incidentally studies focussing on the antibacterial aspect of copper nanowires are very sparse compared to copper nanoparticles. Sànchez-Sanhueza *et al*. [30], reported strong antibacterial activity of copper nanowires on endodontic strains of bacteria and Pinto *et al*. [28] did a comparative study of antibacterial copper nanowire vs nanoparticle on S. *aureus* and K. *pneumoniae* embedded in cellulose matrix. In a separate study, Lin *et al* [31] used pegylated copper nanowires to treat tumour by photothermal therapy using NIR radiation. However, in order to properly evaluate the therapeutic potential of copper nanowires, cytotoxic studies on mammalian cells are an essential preliminary step and this aspect has not been investigated thoroughly. Thus, given their structural difference with nanoparticles, copper nanowire-based composites may enable inexpensive alternative for tissue regenerating scaffolds and wound healing bandages with antibacterial properties.

In this context, with clear aim to consider both cytotoxicity and antibacterial properties, the current work reports a new nanocomposite antibacterial film by embedding CuNWs in a biocompatible and biodegradable hypromellose matrix. We seek to minimize the cytotoxicity of copper by using copper nanowires and evaluate its antibacterial and cytotoxic *modus operandi* for optimum biomedical and therapeutic applications such as antibacterial coatings and bandages for treatment of infectious wounds. Hypromellose is a water soluble and non-ionic polymer which has been widely used as a drug delivery matrix for its swelling and dissolution properties. The rationale of embedding nanowires is that it will reinforce the hypromellose matrix by forming an entangled network leading to slower release. Additionally, the slow release of the high aspect ratio nanowires and biocompatible matrix is expected to be less cytotoxic than copper nanoparticles. In order to highlight this, we have performed detailed biocompatibility studies (cell viability assays and fluorescence imaging) with this film on human dermal fibroblast cell line over 48 h in a concentration dependent manner.

## Materials

Copper (II) chloride (CuCl_2_) (anhydrous, powder, ≥99.995% trace metals basis, Sigma Aldrich), ethylenediamine (EDA) (C_2_H_8_N_2_) (≥99%, Sigma Aldrich), hydrazine hydrate (N_2_H_4_) (reagent grade, 50-60%, Sigma Aldrich), potassium hydroxide (KOH) (reagent grade, 90%, flakes, Sigma Aldrich), (hydroxypropyl)methyl cellulose (HPMC) (average mol wt 10,000, Sigma Aldrich), polyethylene glycol 6000 (PEG 6000) (analytical grade, Sigma Aldrich), polyvinylpyrrolidone (PVP) (average mol wt 360,000, Sigma Aldrich), MTT assay reagents (Vybrant MTT cell proliferation assay kit, Thermofisher Scientific), beef extract, peptone, yeast extract, agar (Sigma Aldrich), Dulbecco’s Modified Eagle Medium (DMEM), trypsin, fetal bovine serum (FBS), penicillin-streptomycin-neomycin (PSN) solution (Sigma Aldrich), Live/Dead assay kit (L3224, Thermofisher Scientific), dichloro-dihydro-fluorescein diacetate (DCFHDA) (>97%, Sigma Aldrich) were used as received.

### Synthesis of the copper nanowires

The synthesis of copper nanowires was carried out following [32], with slight modification. In brief, 64.32g of KOH was dissolved in 80 mL of distilled water taken in a jacketed glass beaker under constant stirring at 85 °C. Next, 400 μL of EDA was added followed by 4 mL of 0.1M CuCl_2_ leading to the solution turning blue. After brief mixing, 110 μL of hydrazine hydrate was added, after which the solution turned transparent and was then left at room temperature without stirring. The solution gradually turned from transparent to deep red and after 15 mins, the copper nanowires accumulated as a layer on top of the mixture. The nanowires were then collected after centrifuging (at 5000 rpm for 10 min) three times with water and finally with isopropanol. They are then stored in 3% hydrazine hydrate solution for further use.

### Synthesis of the HPMC-CuNW films

HPMC-CuNW films were synthesized by a solution casting approach. Initially, 0.1g of PEG 6000 was dissolved in 10 mL distilled water (which was deoxygenated in advance by passing N2 gas for 2 min) under constant stirring at room temperature. Next, 0.03 g of CuNW (dispersed in 0.5% PVP) was added to the solution. The solution was then pulsed sonicated (700 Watt, 3s on and 5s off) for 10 mins and left to stir for another 10 mins at 600 rpm. Thereafter, 0.5 g of HPMC was added and the whole mixture was stirred at 90 °C for 1 h in a capped glass vial. The mixture was then kept at 4 °C overnight to allow for swelling. The film was obtained by pouring the mixture on a plastic petridish and drying at 40 °C. The final CuNW loading in the film was 4.8 wt% maintaining similarity with earlier report [30].

### Characterisation

The absorption spectra of HPMC-CuNW were measured by a UV–Visible spectrophotometer (Lambda 25, Perkin Elmer) in the wavelength range 300–750 nm. A small portion of the film was dissolved in distilled water and then analysed by the spectrophotometer. X-ray diffraction (XRD) patterns of HPMC-CuNW were recorded by a Rigaku, MiniFlex 600 using CuKα radiation (1.5409Å) and scan range (2θ) from 0 to 80° (at 40 kV). Fourier transform infrared spectroscopy (FTIR) (FTIR-8400S, Shimadzu) was performed to determine the bond vibrations of respective phases in the film. Samples were characterized using a diffuse reflectance mode attachment with a scanning range set from 500 to 3500 cm^−1^ under Happ-Genzel configuration.

A field emission scanning electron microscope (FESEM, Zeiss, 1450XB) was used for morphological characterisation. A small portion of the film was directly placed on a carbon coated grid and then sputter coated with gold and observed at 20 kV.

To estimate the dissolution time of the HPMC-CuNW film, 0.1 g of the film was placed in 10 mL of simulated body fluid (SBF) and images were taken at definite intervals at 37 °C. Similar experiments were done by placing the film on an agar plate. Photographic images were taken at specified times (from 0-240 mins) to determine the degradability of the film.

### Antibacterial Study

Antibacterial activity of the HPMC-CuNW film was studied in standard Luria-Bertani broth for *E. coli* (ATCC 25922) and *S. aureus* (ATCC 43300) by colony forming units (CFU) method on agar plates. Typically, 50 mg (for *S. aureus*) and 40 mg (for *E. coli*) of the film were added to cultures of bacteria (taken at a concentration of 10^7^ CFU/mL) in 5 mL nutrient broth (0.5% peptone, 0.1% beef extract, 0.2% yeast extract, 0.5% NaCl, pH 7.4) respectively. The cultures were then incubated at 37 °C on a rotary shaker. Growth inhibition with time was followed by plating 20 μL of the treated cultures on nutrient agar (same composition as nutrient broth but with 1.5% agar as the solidifying agent) at 0, 2, 4, 8, 16 and 24 h. A control was prepared without the film. Bacterial colonies were counted and compared with the control after each interval of incubation. The whole experiment was repeated thrice with fresh film and culture to ensure reproducibility. The antibacterial effect was determined based on cell mortality rate using the equation

(1) $$M(\%)=(\frac{C-B}{C})\;100,$$

where, *M* is the mortality rate (%), *C* is the mean number of bacteria on the control samples (CFU/sample) and *B* is the mean number of bacteria on the treated samples (CFU/sample) [10]. Minimum bactericidal concentration (MBC) values were calculated using the standard plate count technique [18] with increasing concentrations of HPMC-CuNW.

Statistical analysis was done with one-way ANOVA which compares three or more groups defined by a single factor. Differences between groups were assessed by one-way ANOVA using graph pad Instat version 5.0 software. The comparison was done between HPMC-CuNW treated with two different microbial species and their control. Dunnet's multiple comparison tests were performed for intergroup comparisons using the least significance difference tests. A value of *p* < 0.05 was considered to indicate significance. All data were expressed as mean ± standard error of the mean of four separate experiments.

To study the effect of HPMC-CuNW on bacterial membrane, electron microscopy was exploited as follows. Cultures of bacteria in mid exponential phase and with same cell density (as used for antibacterial study) were treated with 50 mg of the HPMC-CuNW film for 6 h at 37 °C. A control was prepared under similar conditions but without the film. The cells were then washed with PBS by repeated centrifugation at 3000 rpm for 5 minutes. Finally, the cell pellet was fixed with 2% glutaraldehyde (in PBS) for 30 minutes. Next, 1 μL of the suspension was placed on a glass cover slip and a series of dehydration steps using increasing concentrations of ethanol (50%, 75% and 100%) in PBS were carried out followed by staining with 3% uranyl acetate in 25% ethanol. Finally, the samples were washed with buffer solution (0.1 M sodium phosphate, pH 7.2) and sputter coated with gold [10] before SEM imaging.

As reactive oxygen species (ROS) generation is one of the major pathways for copper to achieve antibacterial activity, its presence in the HPMC-CuNW treated bacterial solution was determined using DCFH-DA as a fluorescent probe. In a fresh broth (5 mL), overnight grown bacterial culture (*E. coli* and *S. aureus*) were inoculated with a cell density of 10^7^ CFU/mL. The cultures were then incubated with 50 mg of HPMC-CuNW film for 3 h at 37 °C under shaking condition. After incubation, the broth was treated with 10 μL DCFH-DA solution and incubated for further 30 min. Finally, the cells were washed with PBS and the ROS level was measured by a fluorescence spectrophotometer (FluoroMax, Horiba) with excitation at 490 nm and emission at 520 nm [8].

To further understand the antibacterial mechanism of copper nanowires, we determined the amount of copper ion released from the nanocomposite film. Copper ion release from the composite film was qualitatively determined by UV–visible spectroscopy (Lambda 25, Perkin Elmer). A metallochromic dye Alizarin Red S (ARS) was used to bind the released copper ions in the nutrient broth forming a complex with an absorption peak at 510 nm.

Initially, 50 mg of the HPMC-CuNW film was added to each test tube and incubated at 37 °C for 24 h on a rotary shaker. Supernatant from each test tube was collected after 2, 4, 8, 16 and 24 h by centrifugation at 10,000 rpm for 10 min. Next, to each collected supernatant a measured amount of ARS was added from stock (10^−2^ M) along with sodium acetate buffer to maintain an acidic pH. The solution was kept for 10 min and then the optical density (OD) was measured at 510 nm using the UV–Visible spectrophotometer. The intensity of absorption is directly proportional to the amount of Cu–ARS complex which in turn depends on the concentration of Cu^2+^ ions. The dye has a high sensitivity with a detection limit in the range of 0.038 μg/mL. The Cu–ARS reaction is rapid and the complex is stable for up to 24 h [23]. The experiment was carried out three times and reproducible data were obtained.

### Biocompatibility study

Biocompatibility study with HPMC-CuNW was done on human dermal fibroblast (HDF)cell line by tetrazolium dye (MTT) based assay. The cells were cultured in DMEM media supplemented with 10% FBS and 1% antibiotic (PSN) at 37 °C in a humidified atmosphere with 5% CO_2_. After 75–80% confluency, cells were harvested with 0.025% trypsin and 0.52 mM EDTA in phosphate buffered saline (PBS) and were seeded at desired density to allow them to re-equilibrate for a day before the start of experimentation.

MTT [3-(4,5-dimethylthiazol-2-yl)-2,5-diphenyltetrazolium bromide] assay was used to evaluate cell viability as previously described [10]. Briefly, cells were seeded into 24-well culture plate in triplicate (1.3 × 10^6^ number of cells in 400 μL DMEM). Mono layers of cells were treated with increasing concentration (2-12 mg/mL) of HPMC-CuNW dispersed in an appropriate volume of PBS. At the end of the incubation period (i.e., 24 h and 48 h respectively), 40 μL of 5 mg/mL MTT stock solution was added in each well. After an additional 4 h of incubation at 37 °C, the resulting intracellular formazan crystals were solubilized with acidic isopropanol and the absorbance of the solution was measured at 595 nm using an ELISA plate reader (Model: Emax, Molecular device, USA) [10, 23].

Additionally, we also observed the proportion of viable cells after treatment with HPMC-CuNW film by using LIVE/DEAD cell viability assay kit (Thermofisher, L3224). Seeded cells were again incubated with same varying concentration of HPMC-CuNW solution as in MTT assay for 24 h and 48 h respectively. After incubation, cells were washed with PBS twice and 50 μL from the LIVE/DEAD assay stock solution (Calcein AM (2 μM) and Ethidium homodimer (4μM)) were added to each well. Fluorescence images were taken after 2 min under a fluorescence microscope (EVOSM5000).

## Result and Discussion

**Figure 1:**
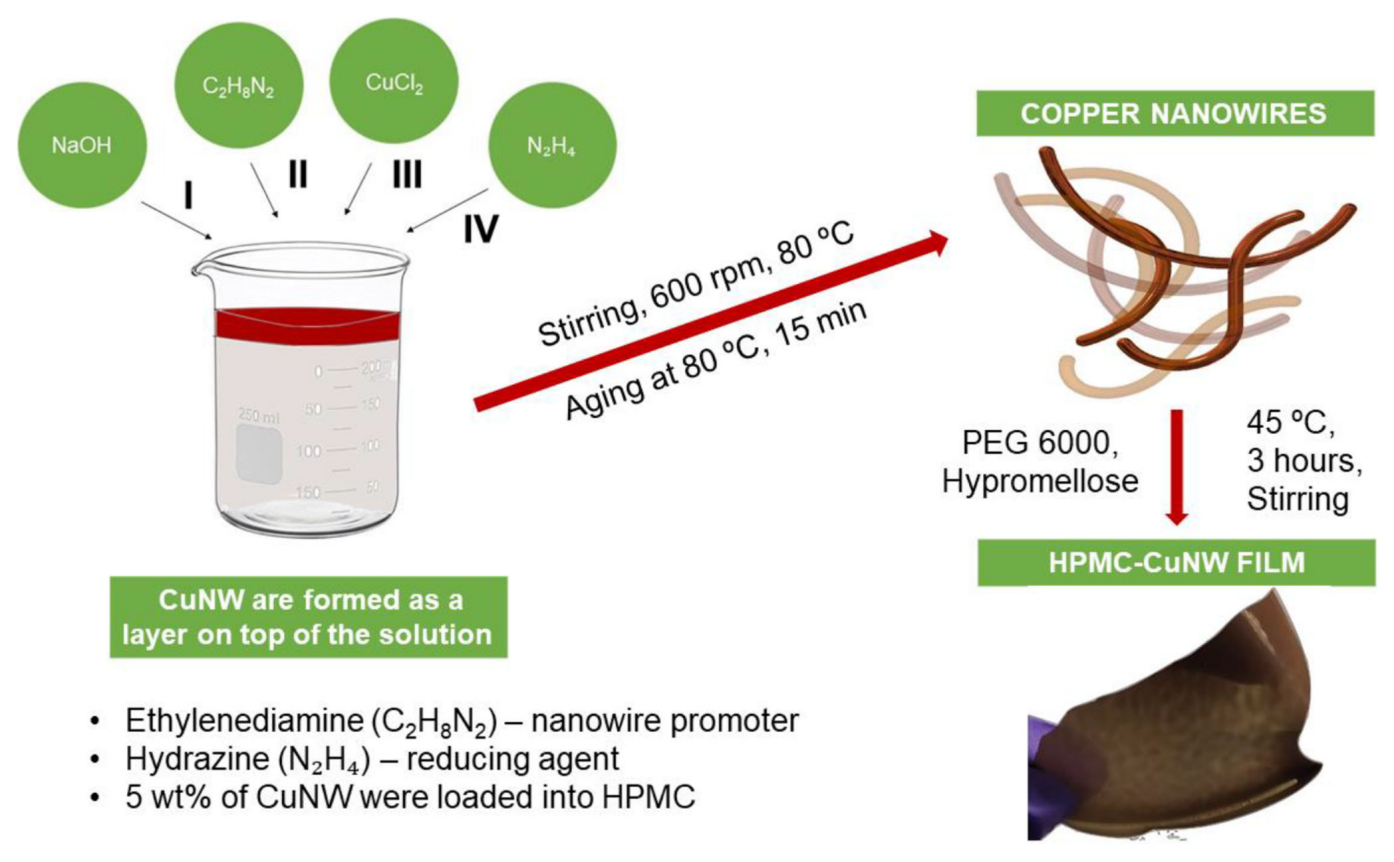
Schematic diagram showing the fabrication of HPMC-CuNW films.

**Figure 2:**
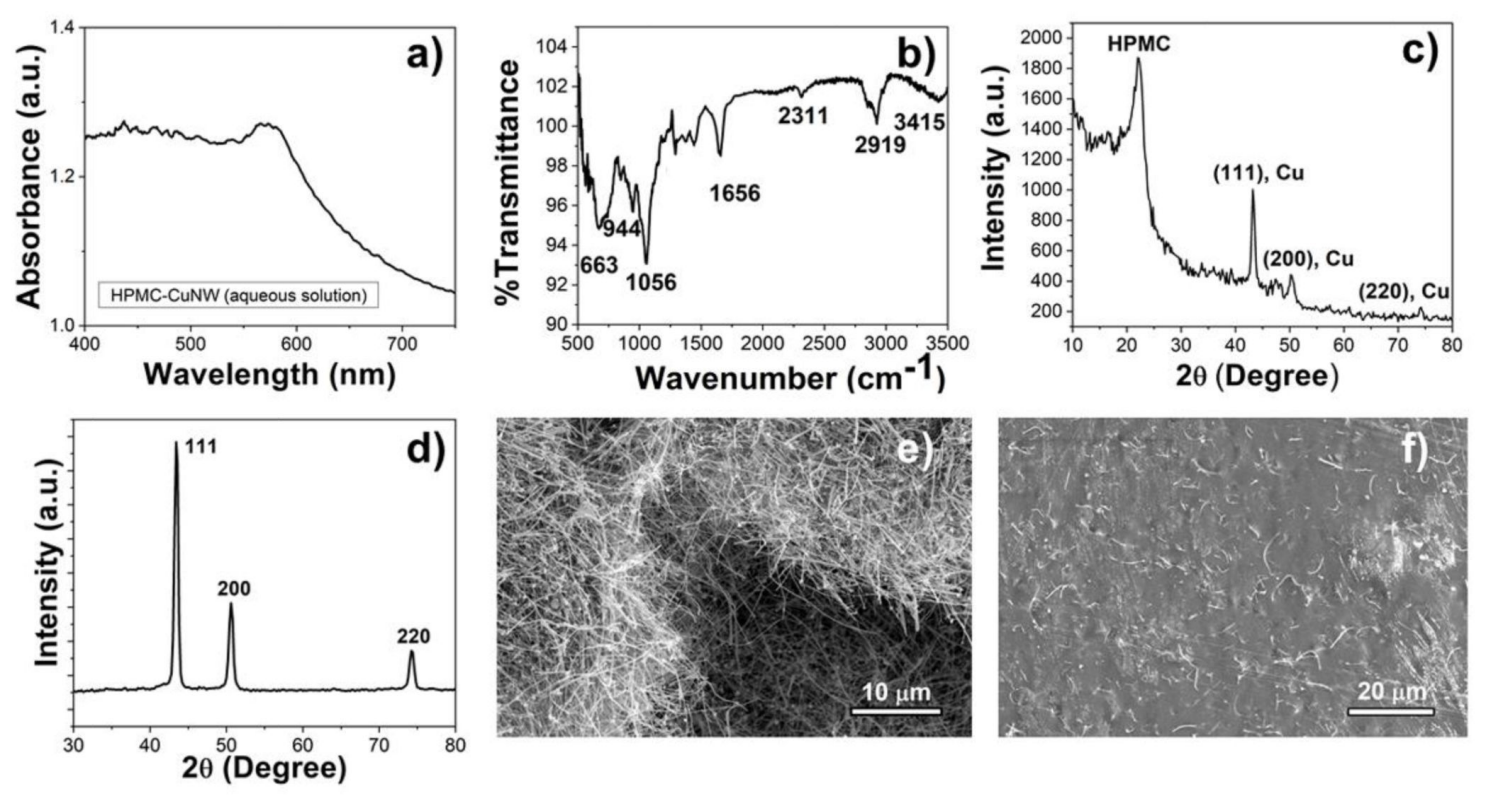
Physico-chemical characterization of the HPMC-CuNW film. a) UV-visible spectra of the film, b) FTIR spectra of the film, c) XRD pattern of the film, d) XRD pattern of the as synthesized copper nanowires and SEM of e) as synthesized CuNW and f) HPMC-CuNW film.

The synthesis procedure of CuNW optimized to get the highest aspect ratio [32]. Figure 1 shows the schematic of the fabrication steps: the CuNWs were initially synthesized followed by their mixing into the HPMC solution and film casting. Figure 1 also shows a photograph of the nanocomposite film. The films formed just by mixing HPMC and CuNW were brittle in nature. In order to counter this, PEG 6000 was added as a plasticiser and its proportion was optimised to get flexible free-standing films. PEG is a well-known and biocompatible plasticiser, which improves the surface texture, flexibility, viscosity and dissolution kinetics of coatings and films in drug delivery applications [33, 34]. Another important factor during the synthesis of the film is the use of deoxygenated water, without which, the solution readily turned greenish yellow during the stirring stage indicating oxidation and hydroxide formation. Properly synthesized films have a deep reddish-brown colour with a smooth glossy appearance and are found to be stable for over two months under ambient conditions. The presence of CuNWs in the film was detected from the characteristic UV-Visible absorption peak around 560 nm (Figure 2a) which corresponds to the transverse surface plasmon resonance band of copper nanowires [35].

Phase composition of the HPMC-CuNW film was analyzed by FTIR spectroscopy. Figure 2b represents the band vibrations of the HPMC-CuNW film. As expected, the film exhibits strong characteristic bands of HPMC at 3415 cm^-1^ (O-H stretching) and 2919 cm^-1^ (C-H stretching), 1656 cm^-1^ (C=O, glucose unit) and 1056 cm^-1^ (C-O-C) [36]. However, all the bands are relatively shifted to lower wavelengths which may indicate intermolecular interaction with CuNW especially through hydrogen bonding [37, 38]. Similarly, PEG 6000 shows bands around 3440 cm^-1^ which overlaps with the O-H group of HPMC and a separate band around 1284 cm^-1^ for C-O stretching mode vibration [39]. Furthermore, some additional peaks were observed around 660 cm^-1^, 944 cm^-1^ and 1400 cm^-1^ which indicate bond vibrations corresponding to N-C=O bending, C-C bond and C-H vibrations of PVP [40]. Again, although distinct bands were observed for each of the components however, shifting of peak positions indicates that HPMC, PEG 6000 and PVP have intermolecular interaction between them as well [37].

X-ray diffraction pattern of the HPMC-CuNW film revealed a broad peak around 2θ = 20° due to the presence of amorphous HPMC. The presence of CuNW is also detected from its characteristic peaks around 43.2°, 50.3° and 74.1° respectively which correspond to a face centered cubic (fcc) structure (JCPDS card no 03-1005) (Figure 2c). The peaks of CuNW are relatively subdued because of the low concentration of copper compared to hypromellose. In contrast, prominent Cu peaks are observed in case of as synthesized CuNWs (Figure 2d). However, no oxide or other peaks were detected in the XRD pattern of HPMC-CuNW which show the stability of CuNW in the film matrix.

Electron microscopy of as synthesized CuNW shows high aspect ratio nanowires with length in the 40-50 μm range and diameter of 50-60 nm (Figure 2e). The microstructure of the HPMC-CuNW film show embedded copper nanowires with an average length of 10-15 μm (Figure 2f), i.e. showing signs of breakage during handling. This shortening of the length has occurred during ultrasonic agitation (as is known for nanowires [41]) to obtain uniformly dispersed nanowires in the HPMC matrix. The final film (see Figure 2f) is nonporous and copper nanowires are predominantly embedded in the film with very few of them being exposed on the surface.

**Figure 3:**
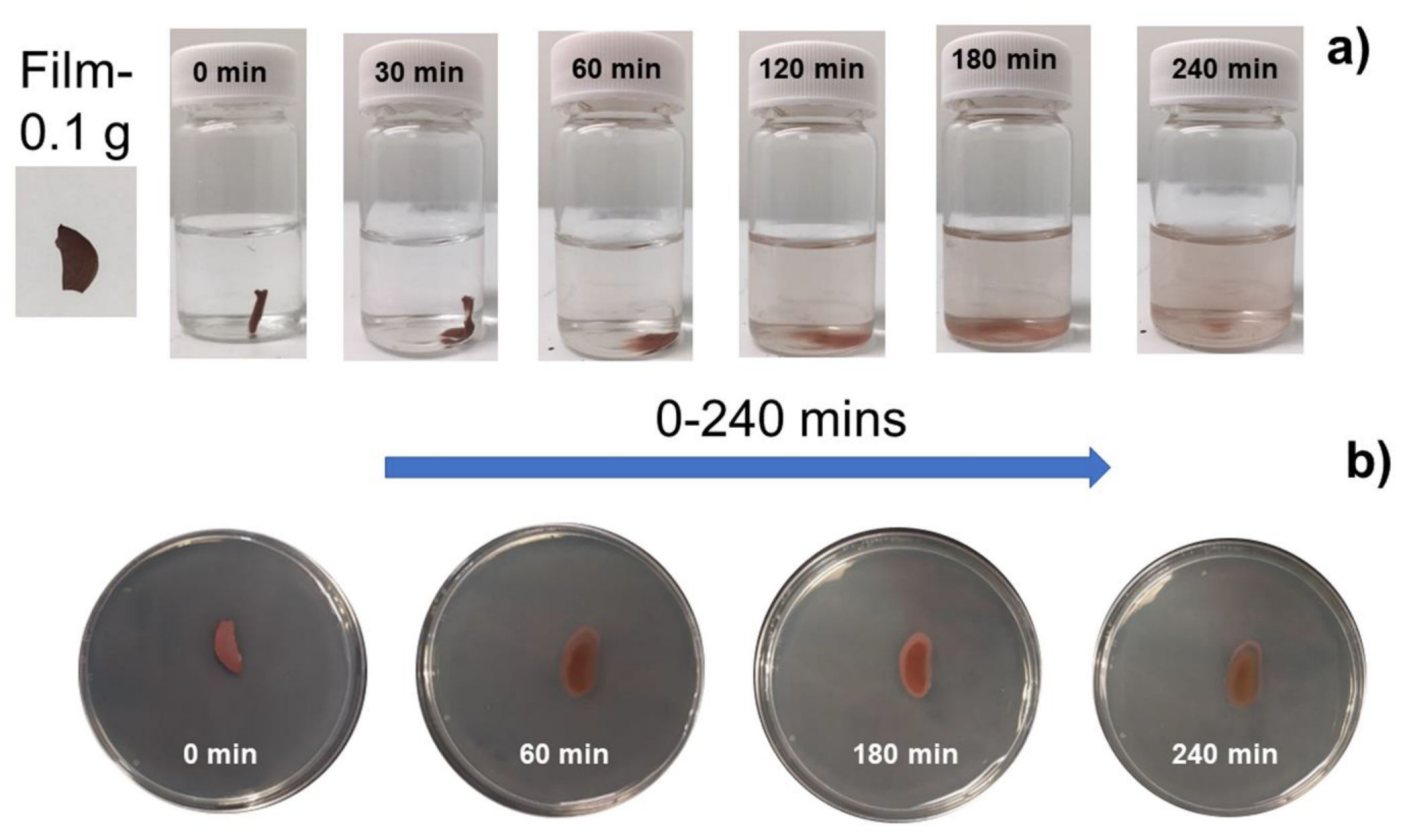
Degradation and solubility study of HPMC-CuNW film in a) phosphate buffered saline (PBS) and b) on agar plates at 37°C.

Figure 3a shows the gradual dissolution of HPMC-CuNW film in PBS. Initially, the film softens, loses its shape and then starts dissolving after 60 minutes without any external/mechanical intervention at 37 °C. The release of CuNW is evident as the solution turns progressively reddish in colour (left to right, Figure 3a). The film completely dissolves after 4 h. In contrast, when the film is placed on agar, it absorbs moisture and diffuses much slower with time and a significant amount of the film remains after 4 h (left to right, Figure 3b). This may be due to the viscous nature of HPMC and PEG 6000 after swelling, resulting in a gel like matrix which slows down the diffusion of CuNW on agar media [33, 34, 39, 42]. Testing the film on agar, which closely mimics a moist and warm environment gives an estimate of the dissolution rate that is pertinent for maintaining antibacterial efficacy during treatment of infected lesions [43]. In both cases, the colour of the treated film does not change, which indicates that the CuNWs are predominantly in their native state without any significant oxidation within the given time period. This is probably due to the protective coating forming due to the interaction between the polymers and CuNW [17–20].

### Antimicrobial study

**Figure 4:**
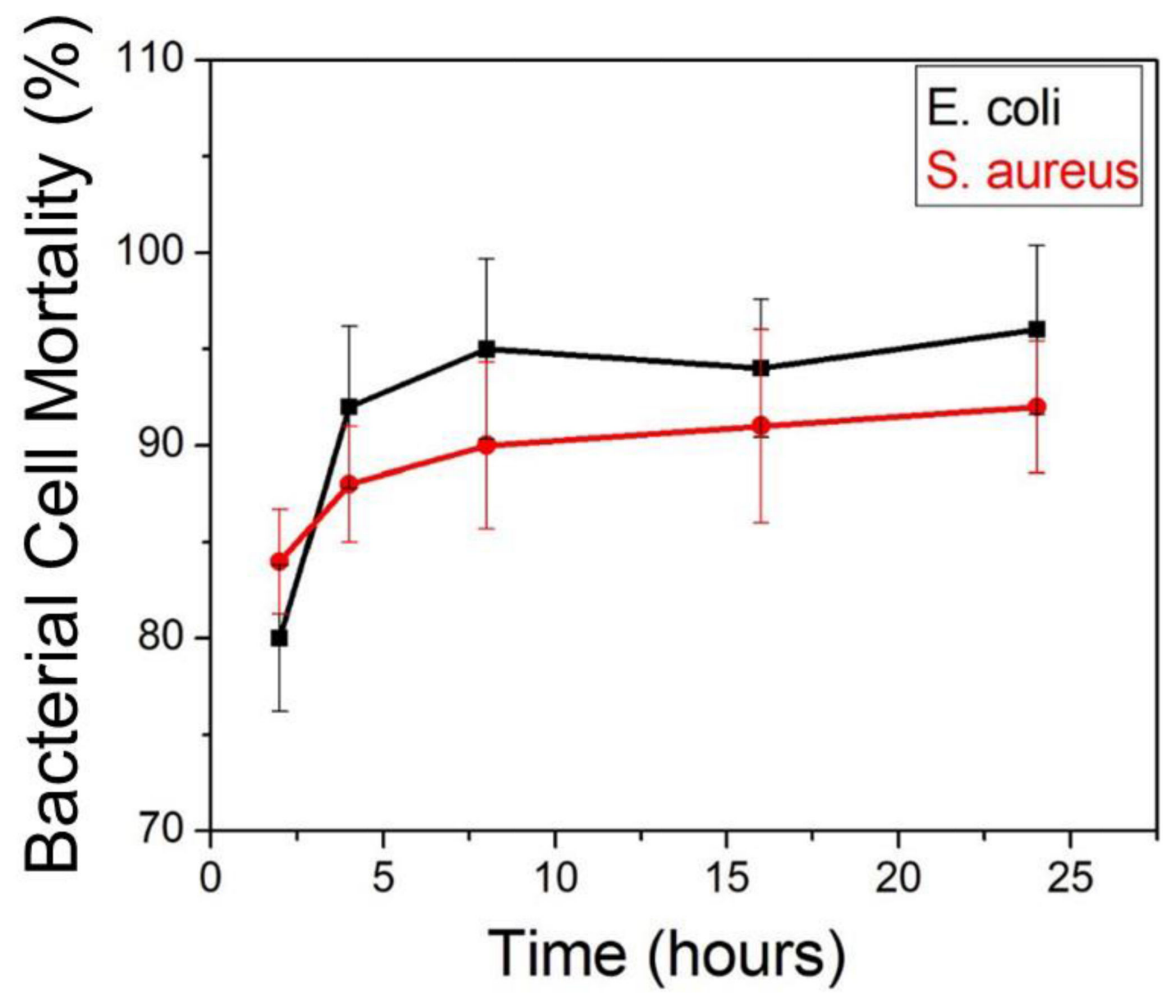
Cell mortality of *E. coli* and *S. aureus* after 24 h of incubation with HPMC-CuNW film. n= 3, p< 0.05.

To investigate the antibacterial activity, bacterial cultures were incubated with HPMC-CuNW (using a concentration slightly lower than MBC) film at different time intervals. After each time, plating was done to determine CFU/mL. The mortality percent of bacterial cells is plotted in Figure 4.

**Figure 5:**
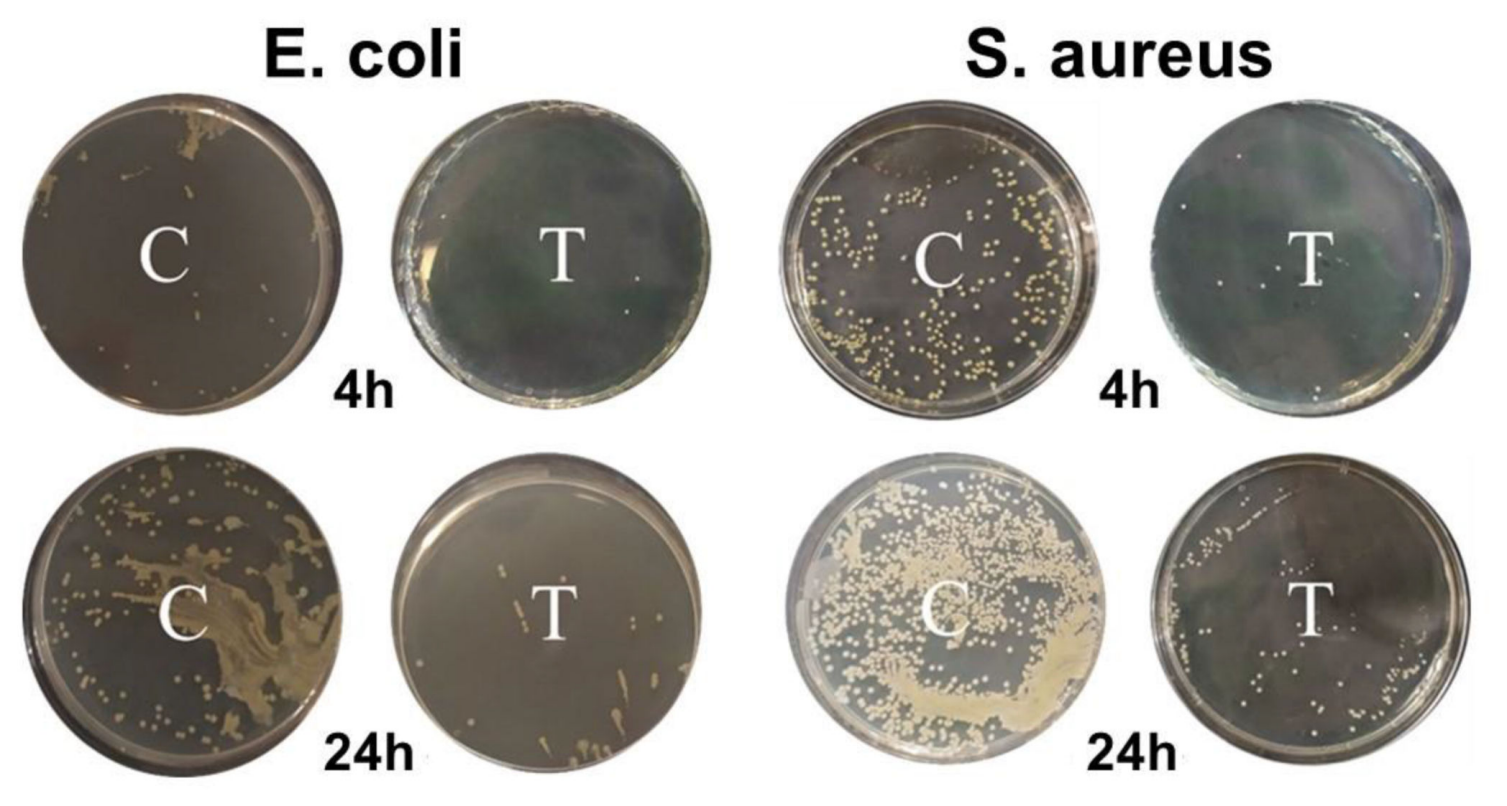
Plate count images showing bactericidal activity of HPMC-CuNW film after 4 and 24 h of incubation.

As evident, for both the *E. coli* and *S. aureus*, cell death percentage becomes significantly higher (≥80%) within 4 h of incubation indicating strong bactericidal effect. Further incubation leads to gradual growth inhibition with *E. coli* being more susceptible (96 % cell mortality) to the bactericidal effect of CuNW after 24 h of incubation. The minimum bactericidal concentration (MBC) value was found to be 400 μg/mL and 500 μg/mL for *E. coli* and *S. aureus* respectively in terms of copper content. Plate images (Figure 5) clearly show difference in colony forming units after 4h and 24 h of incubation and how the number of bacterial colonies is drastically reduced on the treated plate compared to the control (without any film).

**Figure 6:**
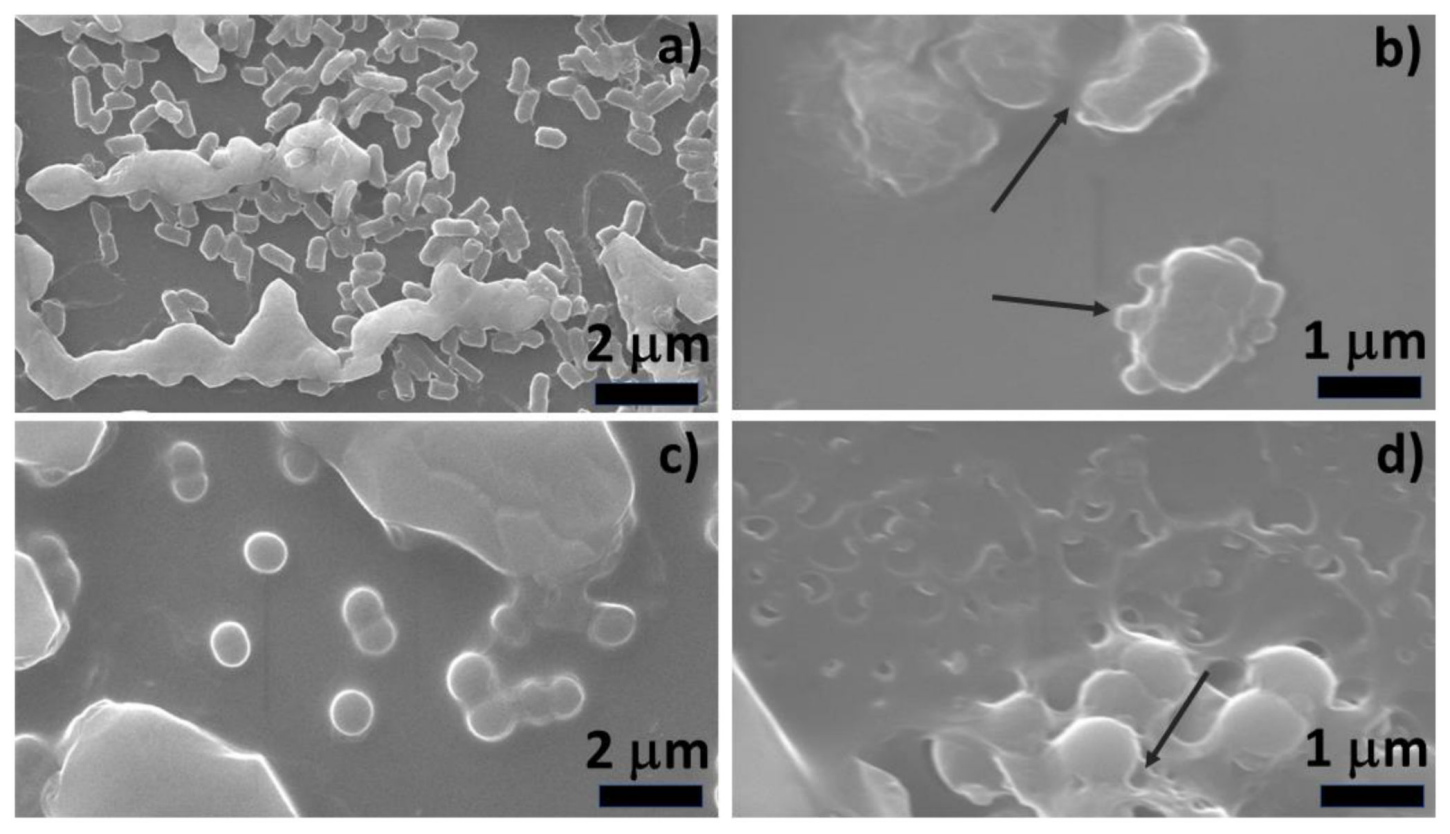
FESEM images of bacterial cells showing morphological characteristics before and after treatment with HPMC-CuNW film for 6 h. a) *E. coli* (control), b) *E. coli* (treated), c) *S. aureus* (control) and d) *S. aureus* (treated). Arrows indicate to damaged cellular membrane.

The bactericidal mechanism of copper nanostructures, especially spherical copper nanoparticles, has been extensively explored and most reports agree that both the metallic copper as well as the released ions are responsible for its antibacterial action [8, 9]. Generally, multiple pathways act simultaneously, causing lipid peroxidation, membrane permeabilization, oxidative stress due to ROS production and protein/DNA damage by Cu ions (released from the nanoparticles) [9, 13]. However, the efficacy of the particles varies widely depending on several factors such as nanoparticle size, shape, coating agent, bacterial strain and growth stage. These interactions lead to membrane perforation, breakdown of the respiratory chain, cytoplasmic leakage and ultimately cell lysis [8, 23, 44, 45]. To examine these effects, we used electron microscopy to observe the morphology of the bacterial cells after treatment with our films (Figure 6). Control images of *E. coli* (Figure 6a) and *S. aureus* (Figure 6c) shows healthy cells with intact membranes. However, treated microbial cells show significant membrane blebbing and rupture followed by cytoplasmic leakage for both *E. coli* (Figure 6b) and *S. aureus* (Figure 6d), typically observed in cells undergoing apoptosis. There is also a drastic change in the shape of *E. coli* where an irregular morphology is observed in contrast to the rod-shaped structure. The observed morphology has been commonly reported in case of copper nanoparticle treated bacterial cells, where pits and cavities on the membrane can be predominantly seen [8. 10, 23]. Thus, the CuNWs clearly possess membrane damaging ability similar to spherical nanoparticles. One of the underlying mechanisms of such membrane perforations has been linked to the damage caused by Cu ions from the nanoparticles. The collapsing of the cytoplasmic membrane leads to morphological changes and shrinkage in the cell. For example, in two different studies, Bagchi *et al* [10, 23] measured Cu ion release from copper nanoparticles embedded in mullite and clay matrices and observed pore formations in both Gram negative and positive bacteria. Later, Chatterjee *et al* [46] also confirmed the role of Cu ions (from copper nanoparticles) in lipid oxidation (which is associated with membrane damage) and DNA damage by using EDTA as a chelating agent. Since, similar membrane damage was observed in case of HPMC-CuNW treated films, therefore, we decided to investigate the release of copper ions from CuNW to determine the rate and concentration of ions with time.

**Figure 7:**
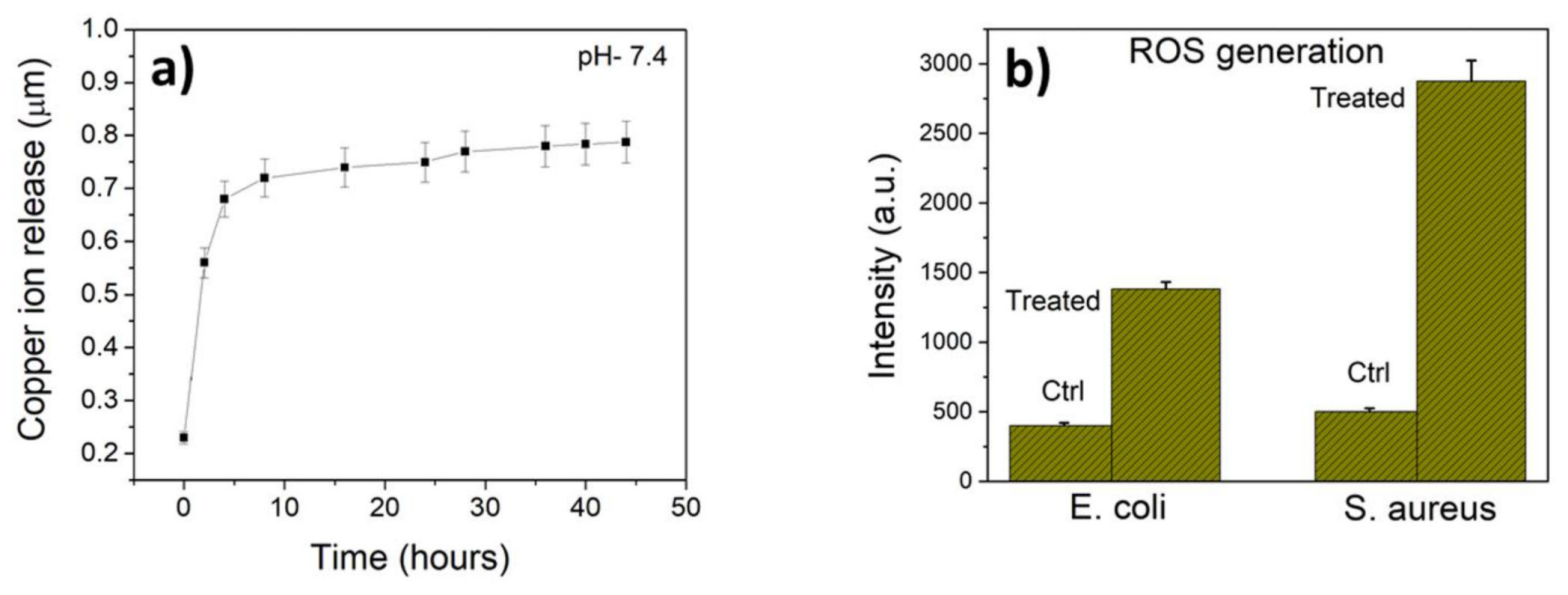
Copper ion release and ROS generation by HPMC-CuNW film

Figure 7a, represents quantitative release of copper ions from copper nanowires over an incubation period of 40 h at 37°C. As evident, the concentration of copper ions steeply rises within 5 h and then gradually increases over a period of 45 h. The initial burst release of ions may come from the fast release of copper nanowires present closer to the surface of the film. However, with time the release becomes slower and more uniform due to the viscous nature of the gradually dissolving film. The highest concentration reached is 0.77 μM at physiological pH of 7.4. Thus, the sustained release clearly highlights the potential bactericidal efficacy of the film over a longer duration. This release trend also coincides with the antibacterial activity observed by plate counting technique where ≥85% cell mortality is obtained within 5 h. In this context, it is noted that *E. coli* is affected more than *S. aureus* (Figure 5). This is expected because the peptidoglycan layer in *E. coli* is negatively charged and therefore able to interact more strongly with the Cu^2+^ ions released from CuNW [9, 28]. In fact, this is also highlighted in the FESEM image, where the membrane of *S. aureus* is comparatively less affected in terms of membrane blebbing and rupture (Figure 6d).

Another source of cellular damage is through uncontrolled ROS generation by metallic nanoparticles by reaction with the oxygen and H2O2 (in the cell environment) to produce O_2_^•−^ and OH^•^ radicals via Haber-Weiss and Fenton type reactions [47]. Specifically, for spherical copper nanoparticles, the level of ROS production is dependent on the size of the nanoparticle and rate of copper ion production [46]. Since copper nanowires are different in morphology and size, this prompted us to measure the level of ROS generated from the HPMC-CuNW film. As can be seen from Figure 7b, the level of ROS generated in the treated cells is three-fold and six-fold more in case of *E. coli* and *S. aureus*, respectively. As discussed by Lemire *et. al*., this observed difference in ROS production may come from the fact that *E. coli* has multiple defense mechanisms against oxidative stress by producing several kinds of ROS scavenging enzymes [48]. This may reduce the observed level of ROS compared to *S. aureus*. However, it may be noted that overall, the mortality percent of *E. coli* is higher than *S. aureus*, which indicates that other cell damaging effects (direct physical damage, membrane pore formation and leakage, protein and DNA damage etc) is predominant here.

**Figure 8:**
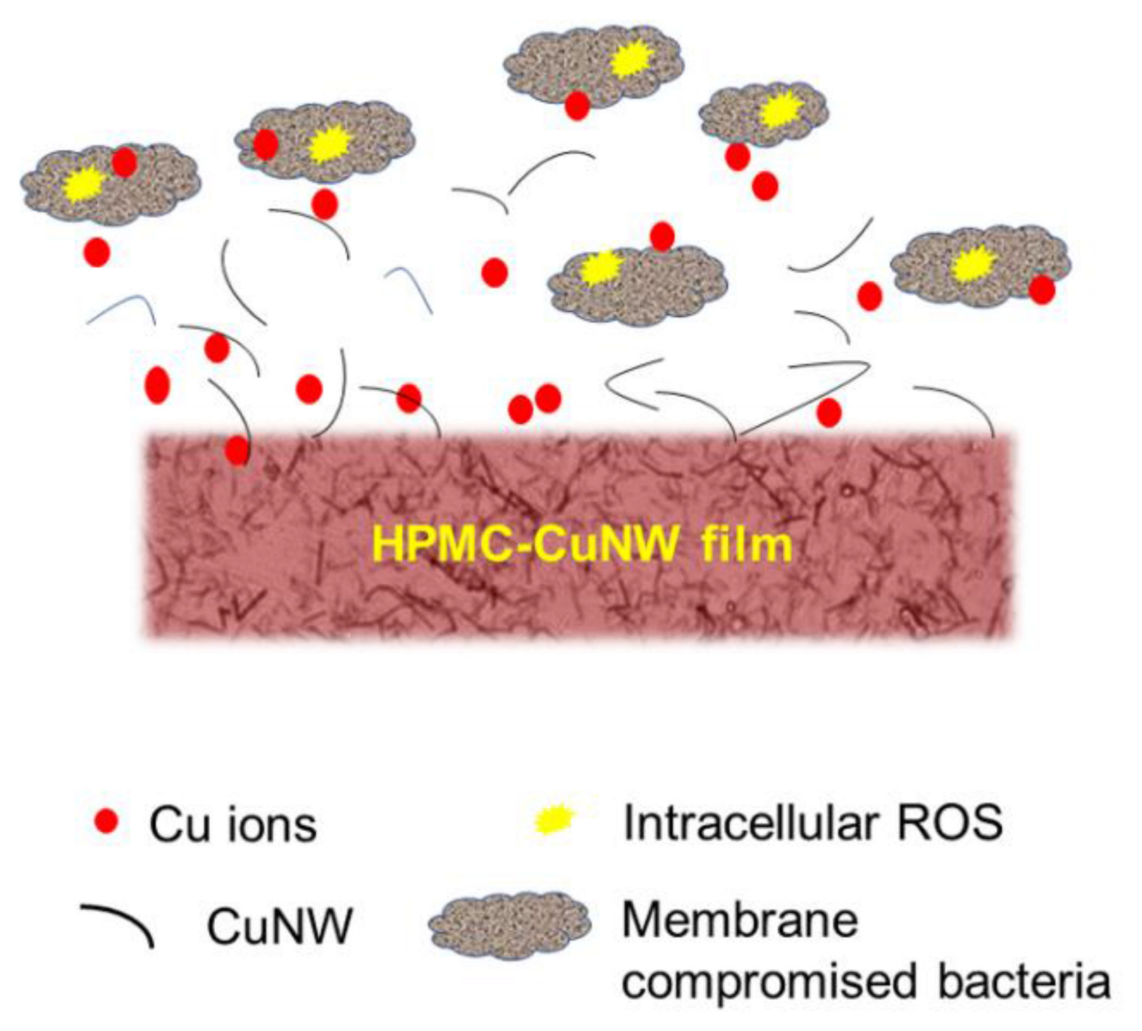
Proposed mechanism of antibacterial action by HPMC-CuNW film.

These observations show that a similar antibacterial mechanism (compared to copper nanoparticles) is at play in case of copper nanowires as shown in Figure 8. Essentially, the idea is that as the film is exposed to moisture, slow dissolution leads to a gradual release of CuNWs from the film and produces Cu^2+^ ions on their surface. Eventually, these Cu^2+^ ions penetrate the cells in the vicinity causing direct membrane damage or ROS mediated damage as described above.

### Biocompatibility study

**Figure 9:**
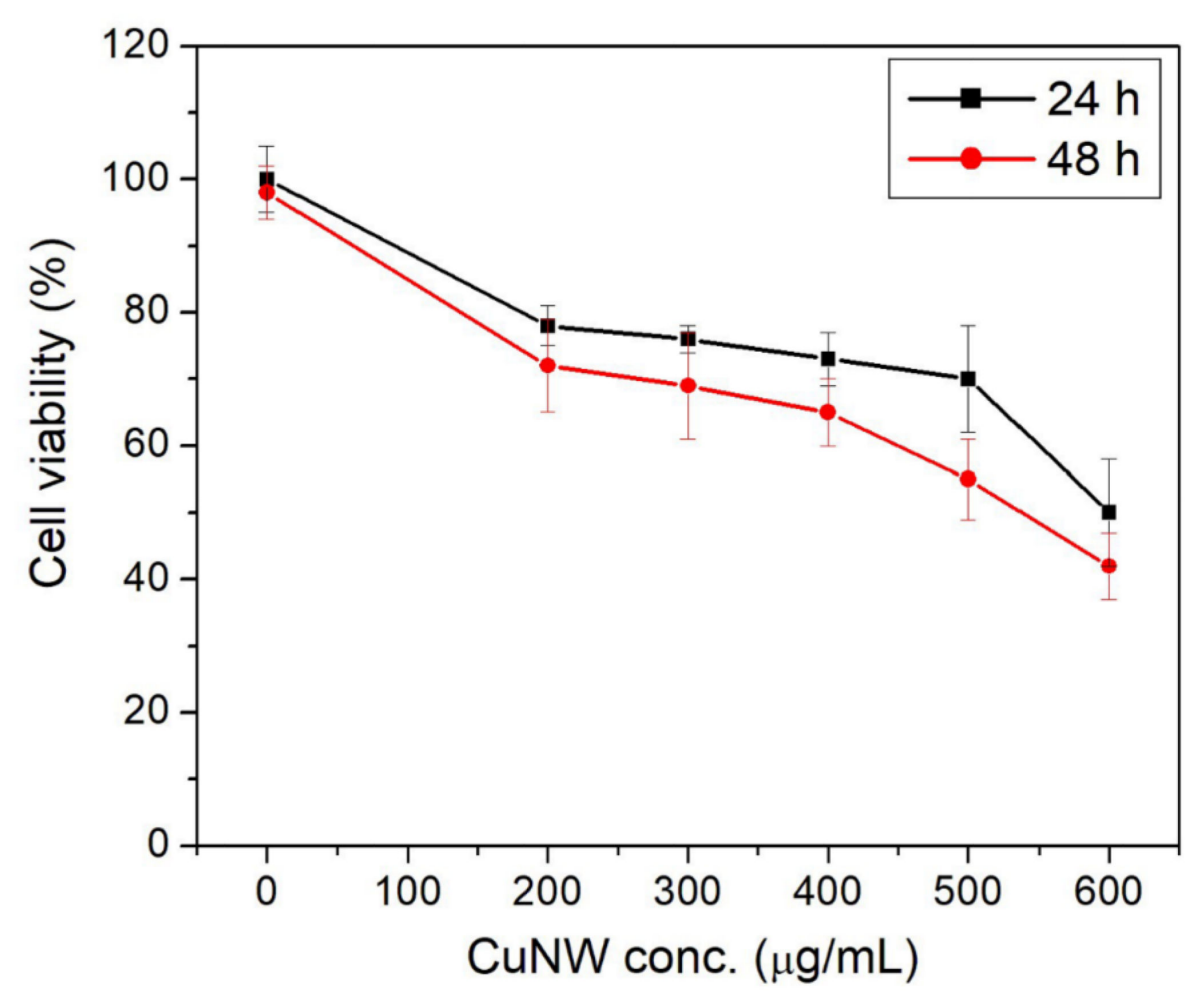
MTT assay showing cell viability of HDF cell lines after treatment with HPMC-CuNW film. n=3, p<0.05.

Biomedical applications of antimicrobial materials require that they exhibit microbicidal effect while remaining reasonably noncytotoxic and in some cases even providing support and stimulation to cells and tissues around the affected region leading to tissue regeneration [49]. It has been shown that the size and dose dependent cytotoxicity associated with copper nanoparticles [50, 51] can be effectively minimised by arresting them in biocompatible matrices, which can than serve as antimicrobial tissue regenerating scaffolds without significant toxicity [24, 52]. Copper nanowires, although, chemically similar to copper nanoparticles, have different morphology, lower surface to volume ratio and hence different ion release kinetics. All of these may have unique effects on eukaryotic cells and may serve as an alternative for spherical copper nanoparticles. Keeping this in mind, cytocompatibility of the CuNW-HPMC films was determined by MTT assay using human dermal fibroblast (HDF, Figure 9) after 24 h and 48 h of incubation. Dermal fibroblasts were chosen as these are actively involved in wound healing by forming new extracellular matrix and regulating growth of healthy cells in the affected area [53].

Figure 9 represents the viability profile of HDF with increasing copper nanowire concentration. In the 200-500 μg/mL concentration range, the viability remains around 75-80%; however, a sharp decline is observed at 600 μg/mL where around 53% of cell death occurred after 24 h incubation. When incubated for 48 h, the cell viability was found to decrease even further from the starting concentration of 200 μg/mL but remained around 55-70% till 500 μg/mL. Again for 600 μg/mL, a significant drop to 42% viability was observed. Notably, the HPMC-CuNW nanocomposite showed reasonably good biocompatibility (≈80% cell viability) in the MIC range (400-500 μg/mL) for both the bacteria. This observation is significant, as in a recent study, Beltran-Pertida *et al* [54] reported much higher cytotoxicity (<25 % viability) with ascorbic acid capped copper nanoparticles on human gingival fibroblasts at a concentration of 500 μg/mL. Earlier studies on cytotoxicity with spherical copper nanoparticles always showed a dose dependent and cell dependent behaviour. For example, Tao *et al* [24] reported minimal cytotoxicity and NIH-3T3 fibroblast proliferation with copper nanoparticle loaded hydrogel. In a different study, Tripathi *et al* [55] also reported moderate biocompatibility of copper nanoparticle containing nanocomposite scaffold on rat osteoprogenitor cells. In contrast, Azizi *et al* [56] observed dose dependent cytotoxicity of albumin coated copper nanoparticles on human breast cancer cells but not on normal cells. The observed high survivability of HDF in the current work focussing on CuNW may be based on multiple factors. First, the morphology of copper nanowires makes it difficult for them to easily enter a cell compared to nanoparticles. Secondly, as already mentioned by Pinto *et al* [28] the rate of Cu ion production and consequently ROS generation are lower in the case of nanowires (compared to nanoparticles), which directly affects cytotoxicity. This is also reflected by the higher MBC value of CuNW observed in case of bacteria. Finally, the new hypromellose-PEG matrix may play an important role in the slow release and thus lowering cytotoxic concentration of ions by forming a gel like environment. Additionally, since both hypromellose and PEGs have been known to promote proliferation of mammalian cells, therefore they may also actively contribute towards the biocompatibility [57–59]. Compared to the antibacterial study, the biocompatibility assay was performed in a much smaller volume of media (400 μL) and thus the local concentration of HPMC and PEG film will be much higher forming a viscous, cell friendly layer. However, we believe that this beneficial effect of the polymers is compromised at higher concentrations of CuNW (>500 μg/mL).

**Figure 10:**
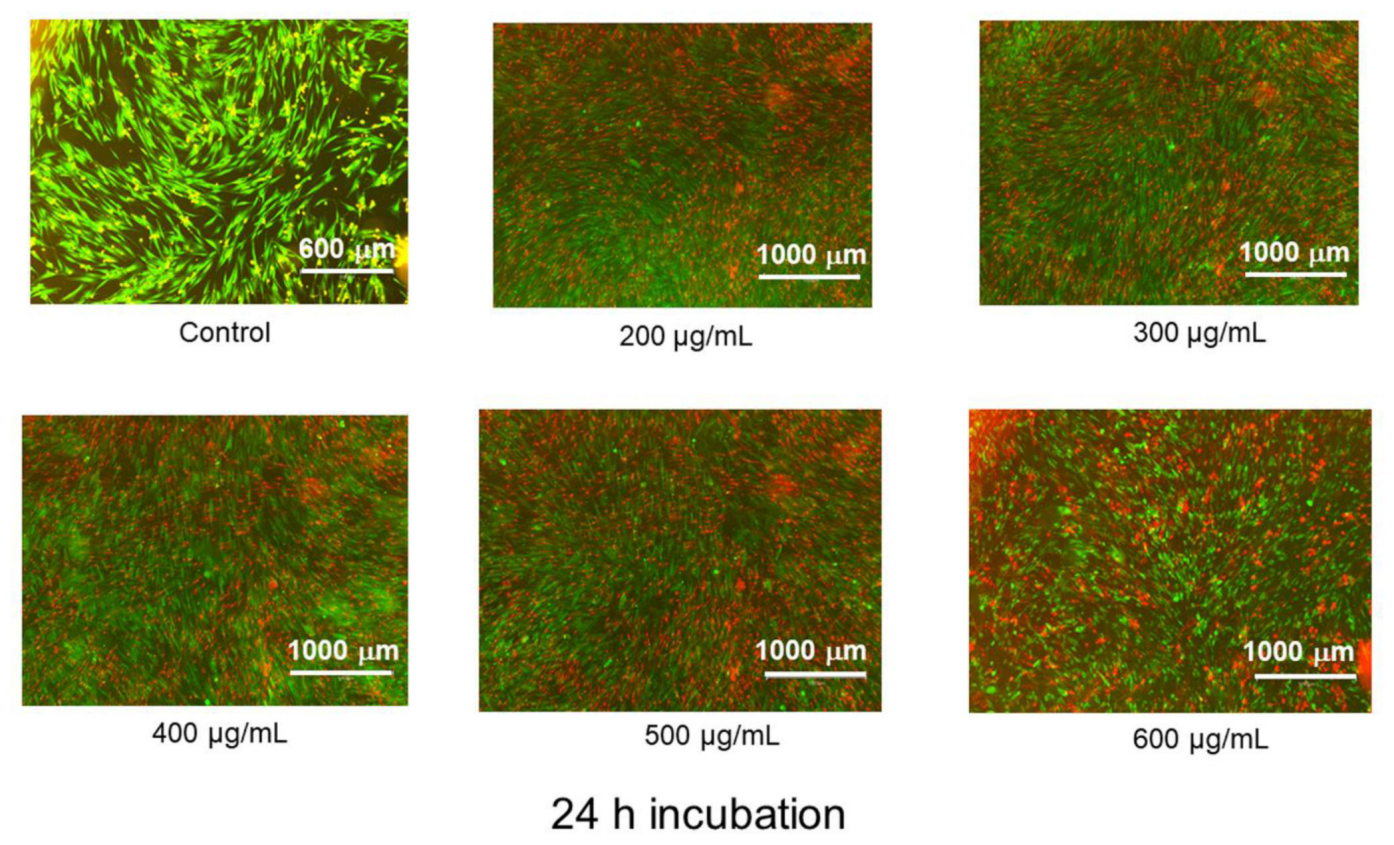
Fluorescence microscopy images of HDF cells after treatment with HPMC-CuNW after 24 h of incubation.

We further confirmed the viability profile with fluorescence microscopy imaging. Figure 10 shows the HDF cells dual stained with Calcein AM and Ethidium homodimer and observed under green and red filter after 24 h incubation.

**Figure 11:**
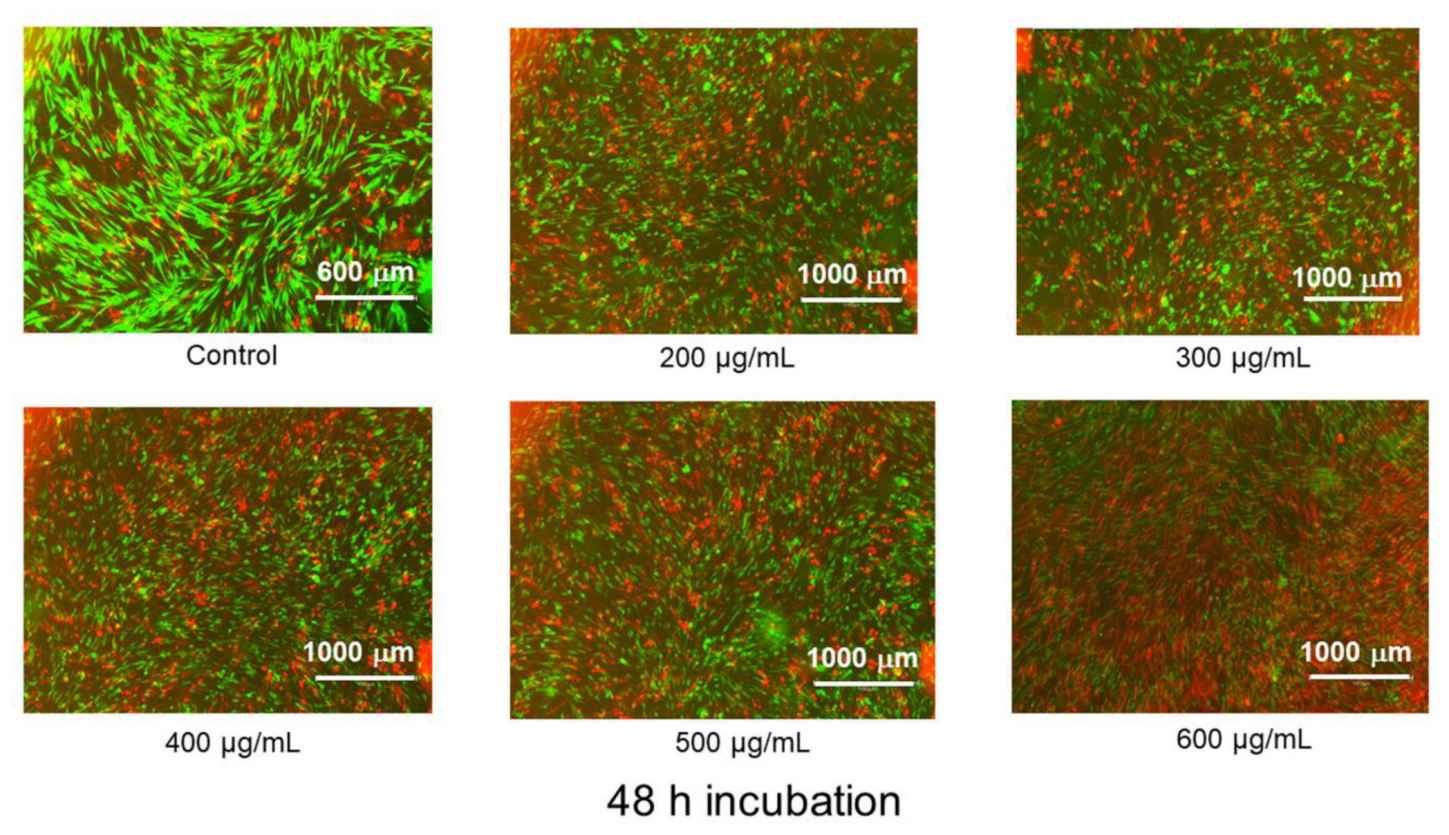
Fluorescence microscopy images of HDF cells after treatment with HPMC-CuNW after 48 h of incubation.

Green spots indicate live cells while red spots represent membrane compromised dead cells [60]. As can be seen from the images (Figure 10), a fairly large proportion of live (green) cells is observed in the 200-500 μg/mL range. At 600 μg/mL, a higher mass of dead (red) cells is present. Similarly, at 48 h, the proportion of live/dead cells is around 50% on going from 200 μg/mL to 500 μg/mL, however, is drastically reduced as the number of dead cells become predominant at 600 μg/mL (Figure 11). Clearly, the CuNW embedded in our nanocomposite is cytotoxic above 500 μg/mL. It is also worth mentioning that the concentration of the CuNW can be easily tailored to achieve an optimum antibacterial activity and biocompatibility. Thus, although further studies still need to be performed to fully understand the interaction and cytotoxicity of HPMC-CuNW film on different cell lines, the nanocomposite film shows promising attributes as a potential therapeutic antibacterial material.

## Conclusion

In summary, a novel copper nanowire based flexible nanocomposite film has been developed using PEG blended hypromellose as a biocompatible matrix. The film showed high antibacterial activity (90-96% cell mortality) against *E. coli* and *S. aureus* after 24 h of incubation period. Through fundamental research, we show that the mechanism of antibacterial action of CuNW is similar to that of copper nanoparticles i.e., based on ions release, ROS production and consequent membrane damage [23, 46]. Oxidation of CuNW is successfully prevented through interfacial interaction with hypromellose and PVP which is confirmed by XRD and FTIR spectroscopy. Additionally, this interaction also facilitates colloidal dispersion of CuNW in the polymer solution which results in a flexible film with uniformly distributed copper nanowires after drying. However, one distinguishing feature is the low cytotoxicity observed at MBC concentration on human dermal fibroblast. The HDFs showed >70% viability at 400-500 μg/mL copper nanowire concentration after 24 h. According to previously published research, copper nanoparticles at this concentration have been found to be quite cytotoxic [54–56], thus raising concerns regarding their therapeutic potential. The observed biocompatibility in the HPMC-CuNW film is probably due to the restricted release and non-penetrability of cells by the high aspect ratio nanowires and the protective role of the viscous polymer matrix. Notwithstanding the fact that the high antibacterial activity, slow dissolution of the film in a moist environment and low cytotoxicity of the HPMC-CuNW film makes it a promising alternative for application such as wound healing bandages and implants, we plan to further undertake a more detailed investigation in future involving different pathogenic strains of microbes and human cell lines to better assess the potential of CuNW based therapeutics.
